# Lectures on Orthopedic Surgery and Diseases of the Joints

**Published:** 1876-08

**Authors:** 


### Bibliographical.

LECTURES ON ORTHOPEDIC SURGERY AND DISEASES OF THE JOINTS; Delivered at Bellevue Hospital Medical College, during the winter session of 1874 and 1875. By Lewis A. Sayre, Professor of Orthopedic Surgery, Fractures and Dislocations, and Clinical Surgeon in Bellevue Hospital Medical Chllege; Surgeon to Bellevue Hospital, etc. Illustrated by 274 wood engravings. D. Appleton & Co., 549 and 551 Broadway, New York. 1876.

The improvement, and we might almost say perfection, to which Professor Sayre has arrived in the treatment of many of the affections of which this volume treats, is truly wonderful. Take, for example, hip-joint disease, which twenty years ago was abandoned by the profession as incurable, thus dooming the poor sufferer to a life of misery and despair; and if by chance nature eliminated the diseased bone, the subject was crippled and deformed for life. Thanks to the distinguished author he has by his labors not only demonstrated the erroneousness of the opinions then held in regard to the character of the disease, but has, by scores of living examples, demonstrated its entire curability, and with limbs so perfect that the casual observer would never for a moment suspect any defect.

For many years the profession, not only in the United States, but in Europe, have urged Dr. Sayre to present his views on Orthopedic Surgery and disease of the joints, and particularly the latter, in a form that they could be referred to at will. No work for years has been received by the profession with so hearty a welcome as this volume, and we predict that this welcome will be increased and strengthened as the contents of the volume become better known.

We regret that our space will not permit us to call attention to the prominent features of the work. We cannot, however, close this notice without giving, in Dr. Sayre's own language, the etiology of hip-joint disease. It was Dr. Sayre's peculiar views as to the cause of hip-joint disease that induced him to attempt its cure, and with the brilliant results so well known. In giving the etiology of morbus coxarius, Dr. Sayre says:

"Almost all surgical authorities agree that morbus coxarius is invariably the result of a contaminated constitution; in other words, that it is essentially strumous in its origin. This has been the universal opinion, and the doctrine has descended from teacher to student, and is still extant among the majority of surgical practitioners. It has been so often taught and enforced by frequent repetitions, that nobody considered it worth while to question its truth; but nearly all have taken it for granted that an assertion so positively made and universally accepted must be based on mature investigation. When I first entered the profession I accepted this doctrine taught by our fathers, but must confess that I never was fully satisfied with regard to its correctness. Now, while I revere the labors of those great men in the advancement of scientific investigation, I must be permitted to question what is questionable, and to doubt what is doubtful.

"Examination of the cases which have presented themselves to my notice since that time, has convinced me that the cachetic condition so often seen is the *result* and not the *cause* of the disease; for very many of the patients in the earlier stages of the disease have possessed all the appearances of robust health, and in all those cases in which the disease has been cured by nature's method, the patient, subsequent to the cure, has been hale and hearty. I do not suppose there is a person in this room who cannot call to mind some old fellow with a shortened hip, perfectly anchylosed, who yet has a ruddy face, a good healthy complexion, and is a vigorous, robust old man. If he had had scrofula in his system, it would have remained there, and when his hip had recovered the man would have been a miserable old fellow after all. The very fact of his becoming a vigorous, robust man after going through all the exhausting effects of hip-joint disease, proves, in my judgment, that the disease is not of constitutional origin.

"The additional fact that, in so many cases, the joint has been exsected when the patients have been, apparently, at the point of death, and after the removal of the dead bone have become vigorous, strong persons, is good evidence that the disease is not constitutional. Then there is the still stronger fact that, by treating the disease locally, without reference to constitutional taint, we obtain perfect results, so much so that the patients recover with perfect motion and without the slightest deformity, which is the best proof in the world that the disease is essentially local in character.

"Another fact worthy of consideration is that a very large proportion of cases of the disease occur in children, while the scrofulous condition is by no means so restricted.

"I have unfortunately recorded only a small part of the cases which have fallen under my observation, but three hundred and sixty-five cases have been fully entered upon my record, and of these two hundred and twenty-one were under the age of fifteen years, and one hundred and twenty-one were under the age of five years. Similar results have been obtained by other gentlemen who have collected statistics upon this point.

"Now, it is not necessary for me to prove that adults are nearly as liable to be affected with scrofulous diseases as are children, the less number of cases seen being due mainly to the fact that these sickly children are very liable to die before reaching adult life. If, therefore, we still adhere to the scrofulous theory, we are forced to conclude that the diathesis, which in childhood develops itself in joint-disease, manifests itself in some other way after puberty. This I cannot believe. Childhood is the age of restless activity, and out of the hundreds of cases in which I have taken the trouble to trace their history, I have found that the immense majority—I may safely say seventy-five per cent.—have occurred in the most vigorous, robust, wild, harum-scarum children—those who take their chances of danger; who run races, climb over fences, jump out of apple-trees, kick their playmates down stairs, ride down balusters, and are generally careless and reckless.

"On the other hand, the adult does not place himself in the position in which he can receive so many blows or falls as the active child does; and furthermore, he immediately notices the effects of his injury, and takes precaution against its development into serious trouble. The child, however, knows nothing of results, and unless the pain from the injury is great, will probably fail to complain of it, and soon forget it altogether. This, I believe, is the true reason why so many more cases of joint-disease are seen in children than in adults.

"I do not wish to be understood as saying that scrofula is a *preventive* of disease of the hip-joint, as has been asserted concerning my teaching. All things considered, a smaller amount of injury will produce the disease in one of these miserable, sickly children, than in a healthy, robust child. But the sickly, scrofulous child, who clings to his mother's apron, does not run the risk of getting hurt as do these active, restless children; consequently the majority of cases occur among the active and robust.

"From what has been said, you have probably already drawn the inference that I regard the disease as one almost invariably due to *traumatic* cause, and *not* dependent upon some constitutional taint. To what has already been said upon this point, we may add the positive evidence of statistics.

"Of the three hundred and sixty-five cases alluded to above, *traumatic* cause was assigned by the patient or the parent in two hundred and fifty-seven, while in one hundred and eight cases the cause was recorded as unknown.

"In two hundred and seventy-eight cases, the previous general condition of the patient was good; in forty-two cases it was bad; and in forty-five cases it was unknown. These figures are taken from the notes of my own fully-recorded cases. Cases not fully recorded have been rejected in making these statistics.

"Now, the cases in which the previous condition was bad, together with those in which it was unrecorded, make up less than twenty-four per cent of the whole; and it is possible that very many of those had a traumatic origin that had been overlooked or forgotten, owing to the insidious manner in which the changes had come on.

"My own clinical observations with reference to this point stand by no means isolated. The same observations have been made by other surgeons, both in this country and Europe.

"It generally requires a very close examination to find out the cause, since the disease does not usually immediately follow the injury, but often first manifests itself weeks, and even months, after the accident that has given rise to it has occurred; so that the patient and his friends naturally enough forget the accident and its connection with the disease, until especially reminded of it in the investigation."

We feel that every practitioner should have this work.

